# The Booster Effect of a Single Quarterly Dose of Hyaluronic Acid in Knee Osteoarthritis: Five-Year Results of a Registry-Based Study

**DOI:** 10.7759/cureus.31592

**Published:** 2022-11-16

**Authors:** Felice Galluccio, Yasser A Gazar, Ahmed A Negm, Mario Fajardo Perez, Ece Yamak Altinpulluk, Tolga Ergönenç, Ke-Vin Chang, Jen L Pan, Abdallah El-Sayed Allam

**Affiliations:** 1 Rheumatology and Rehabilitation, Fisiotech Lab Studio, Firenze, ITA; 2 Pain Medicine, Morphological Madrid Research Center (MoMaRC), Madrid, ESP; 3 Center for Regional Anesthesia and Pain Medicine, Wan Fang Hospital, Taipei Medical University, Taipei, TWN; 4 Rheumatology, Al-Azhar University, Cairo, EGY; 5 Rheumatology and Rehabilitation, Al-Azhar University, Cairo, EGY; 6 Anesthesiology Research Office, Ataturk University Medical School, Erzurum, TUR; 7 Outcomes Research Consortium, Cleveland Clinic Foundation, Cleveland, USA; 8 Anaesthesiology, Akyazi State Hospital, Sakarya, TUR; 9 Physical Medicine and Rehabilitation, National Taiwan University Hospital Bei-Hu Branch, Taipei, TWN; 10 Physical Medicine and Rehabilitation, National Taiwan University College of Medicine, Taipei, TWN; 11 Physical Medicine and Rehabilitation, Taipei Medical University Hospital, Taipei, TWN; 12 Regenerative Medicine, PAN Regenerative Pain Clinic, Taipei, TWN; 13 Physical Medicine, Rheumatology, and Rehabilitation, Tanta University Hospital, Tanta, EGY

**Keywords:** knee injection, booster effect, viscosupplementation, hyaluronic acide, knee osteoarthritis/ koa

## Abstract

Objective

Viscosupplementation by hyaluronic acid (HA) is well established non-surgical treatment of knee osteoarthritis (KOA). This registry-based study investigated the booster effect of a quarterly intra-articular single knee injection (30mg/2ml) for five years.

Methods

Sixty patients, including 29 males and 31 females, with a mean ± SD age 61.07 ± 9.15 with Kellgren-Lawrence grade I-III KOA, have been selected from a registry of interventional treatments for musculoskeletal pain conditions. To be eligible, patients had to be treated with a single quarterly intraarticular injection of HA with a follow-up of at least five years and assessed with Western Ontario and McMaster Universities Arthritis Index (WOMAC) and Numeric Rating Scale (NRS) at baseline and after each HA injection in the first 24 months and at 36, 48, and 60 months.

Results

Sixty of 63 patients enrolled in this study completed the 60 months of follow-up. Patients had a marked improvement in knee function and pain, expressed by the significant reduction in WOMAC (T0 48.62±8.95 vs. T11 10.75±4.36; p<0.0001) and NRS scores (6.38±1.06 vs. T11 0.95±0.89 p<0.0001) from the baseline to the end of the follow-up period.

Conclusion

A quarterly injection of HA provides a rapid, safe, and stable long-term reduction of pain and improvement of function in elderly people with mild to moderate knee osteoarthritis along a five-year period of treatment and follow-up. Further investigations are necessary to confirm these findings.

## Introduction

Osteoarthritis (OA) is the most common joint disease involving all joint structures, including the subchondral bone, ligaments, capsule, synovial membrane, and the periarticular muscles, producing pain, disability, and limiting activities of daily living [1]. The knee is one of the most frequently affected joints, and the burden of knee osteoarthritis (KOA) increases with the advancement of age and obesity [2].

To date, the treatment of KOA aims to delay progression, relieve pain, and improvement of the activities of daily living. Unfortunately, there are no curative treatments but only symptomatic, with prosthetic replacement in the most advanced cases, which is a significant cost to health systems [3]. Treatment includes nonsteroidal anti-inflammatory drugs (systemic and topical), acetaminophen, disease-modifying OA drugs, and symptomatic slow-acting drugs for osteoarthritis [4-7], intra-articular injection of corticosteroids, hyaluronic acid [8-9], or platelet-rich plasma [10], botulinum toxin [11-12], genicular nerve block [13], cooled or traditional radiofrequency ablation [14] and genicular artery embolization [15].

Hyaluronic acid (HA) is a glycosaminoglycan of the synovial fluid and cartilage matrix synthesized by synovial cells, fibroblasts, and chondrocytes. HA has a good safety profile characterized by limited local and systemic side effects and has no drug interactions, making it a very safe treatment in KOA [8].

There are numerous HA formulations on the market, with different molecular weights, from low (range 500,000-730,000 dalton), to intermediate (range 800,000-1,200,000.00), to high molecular weight (> 1,500,000 dalton), and to cross-linked and polymerized with very high molecular weight (up to 6,000,000 dalton). The treatment schedules are also very variable, from weekly injections for three or five weeks every six months, to single doses, to those with a booster after 15 days or one month, and so on. To date, in fact, in the literature, there is no univocal therapeutic protocol for treatment with hyaluronic acids, and therefore the choice is often left to the experience of the doctor or to the clinical response of the patient [9].

This data analysis aims to evaluate the long-term (five years) booster effect of a single quarterly intra-articular injection of a HA, Hyalubrix® (Fidia Farmaceutici S.P.A., Abano Terme, Italy) 30 mg / 2 ml, in KOA. Hyalubrix (30 mg/2 ml) is obtained by fermentation with a molecular weight in the range of 1500-2000 kDa and commonly injected intraarticularly once weekly for three weeks and can be repeated after six months [10-17].

The hypothesis underlying this real-life study in a population with KOA is to evaluate whether a booster administration of HA, repeated at regular intervals, can guarantee a continuity of positive effect on the joint, pain, and functional limitation.

## Materials and methods

Patient selection

This is a single-center, registry-based data analysis of a cohort of patients with KOA treated with a quarterly booster injection of HA and with a follow-up period of 60 months from the osteOArena registry (electronic registry of interventional treatments for musculoskeletal pain conditions) of the first author, from 2015 to today.

To be eligible for selection in this registry-based studio (inclusion criteria), patients had to be diagnosed with a grade I-III KOA (Kellgren-Lawrance staging classification) and treated with a quarterly single intraarticular injection of HA with a follow-up of at least five years and with informed consent to knee injections, data collection, and publication. Ethics board approval was not required for registry-based studios on a labeled procedure and anonymized data in accordance with the policy of our institution. 

The exclusion criteria were knee surgery, recent knee trauma, lower limb length discrepancy, an intra-articular injection with steroids, other HA or any other intraarticular treatment, and/or current/regular treatments with steroids or non-steroidal anti-inflammatory drugs (NSAIDs) within the previous six months.

Treatment

All patients received a full course of treatment with a weekly injection of HA (Hyalubrix) for three consecutive weeks (T0) and then a single booster injection every three months until completing the fifth year of follow-up (T1-T11). No other drugs, steroids, or local anesthetics, are combined with HA.

All the injections were performed under ultrasound guidance with a 3-12MHz linear probe and with a 21Gx2" (0.8 x 50 mm) needle from the superolateral access, with the knee in slight flexion, with sterile disposable material (gloves, gauzes, probe covers, and needles), and dual skin disinfection with chlorhexidine and iodopovidone (10% alcoholic solution). After each injection, patients were asked to limit heavy painful activities for the next 24 hours; local rest and ice packs were allowed if there is a local reaction at the injection site.

Assessment and safety

Patients were assessed by Western Ontario and McMaster Universities Arthritis Index (WOMAC) and Numeric Rating pain Scale (NRS) performed at baseline (T0) and after each HA injection in the first 24 months (T1-T8). In the following months, these two items were calculated at 36 (T9), 48 (T10), and 60 (T11) months of follow-up. Complications were recorded with particular attention to local pain reactions at the injection site and post-injection reactions such as effusion or pseudo-septic and septic arthritis.

Statistics

To evaluate the association between WOMAC and NRS scale and time from infiltration due to the repeated measures on each patient, a Generalized Estimation Equation (GEE) linear regression model was used.

## Results

Sixty-three patients (31 males, 32 females) were selected for this study. The mean age was 61.07±9.15, with a mean BMI of 22.075±2.42. At baseline, 46.6% of patients were in Kellgren-Lawrence (K-L) grade I, 26.6% in K-L II, and 26.66% in K-L III. None of the patients used walking aids. Most of the patients had an active life, most of which still in work.

From the preliminary analysis of the patients included in the registry, 63 patients met the inclusion criteria. Of these, three patients were excluded because they did not reach 60 months of follow-up because of spontaneous osteonecrosis (one patient) and accidental knee injury (two patients).

**Table 1:** Patient's baseline characteristics

	Total	Kellgren-Lawrence 1	Kellgren-Lawrence 2	Kellgren-Lawrence 3
Patients	60	28(46.66%)	16(26.66%)	16(26.66%)
Age	61.066±9.151	60.821±9.899	64.437±8.64	58.125±7.623
Male	59.310±9.4	58.066±9.91	63.375±9.395	57±7.668
Female	62.709±8.745	64±9.246	65.5±8.315	58.8±7.8
Sex (male/female)	29/31	15 (25%)/13 (21.66%)	8 (13.33%)/8 (13.33%)	6 (10%)/10 (16.66%)
BMI	22.075±2.422	21.864±2.777	22.293±2.388	22.225±1.824
Male	22,079±2,89	21.773±3.331	22.837±3.034	21.833±1.306
Female	22,070±1,927	21.969±2.094	21.75±1.530	22.46±2.105

**Figure 1:**
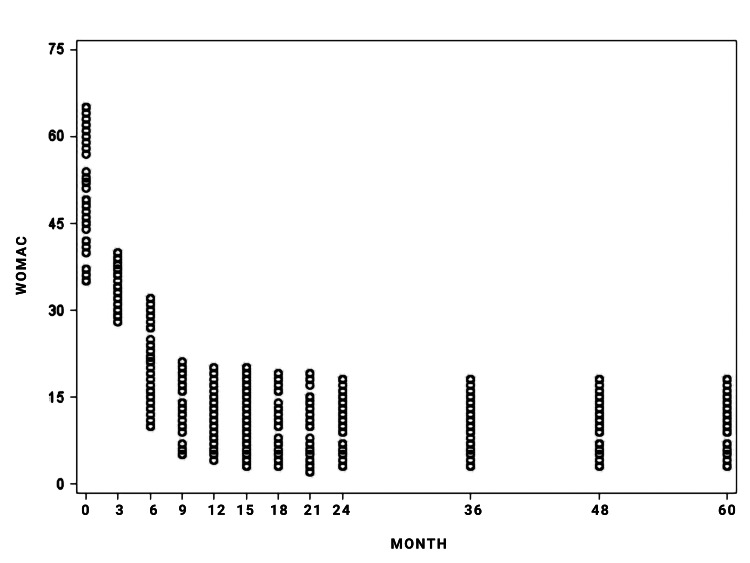
Observed Western Ontario and McMaster Universities Arthritis Index (WOMAC) values

**Figure 2:**
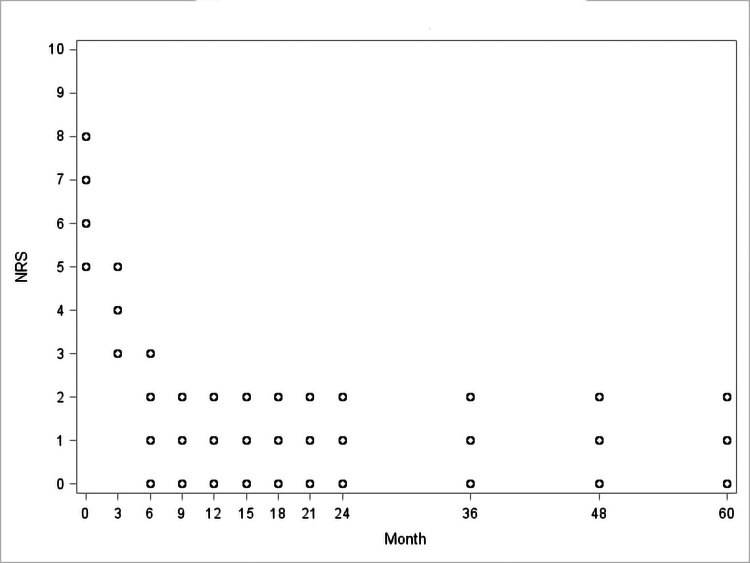
Observed Numeric Rating Scale (NRS) values

Patients had a marked improvement in knee function and pain, expressed by the significant reduction in WOMAC (T0 48.62±8.95 vs. T11 10.75±4.36; p<0.0001; Figure 1, Table 2) and NRS scores (6.38±1.06 vs. T11 0.95±0.89 p<0.0001; Figure 2; Table 2) from the baseline to the end of the follow-up period. 

**Table 2:** Western Ontario and McMaster Universities Arthritis Index (WOMAC) and Numeric Rating pain Scale (NRS) during the five-year follow-up

	T0	T1	T2	T3	T4	T5
WOMAC	48.62 ± 8.95	34.55 ± 3.81	20.90 ± 6.72	13.92 ± 4.97	11.78 ± 4.55	11.91 ± 5.49
NRS	6.38 ±1.06	3.68 ± 0.75	1.53 ± 1.16	0.87 ± 0.87	0.73 ± 0.82	1.04 ± 0.81
	T6	T7	T8	T9	T10	T11
WOMAC	10.15 ± 5.31	9.57 ± 5.35	10.72 ± 4.38	10.73 ± 4.37	10.72 ± 4.38	10.75 ± 4.36
NRS	1.03 ± 0.80	0.92 ± 0.85	0.93 ± 0.88	0.95 ± 0.89	0.93 ± 0.88	0.95 ± 0.89

**Figure 3:**
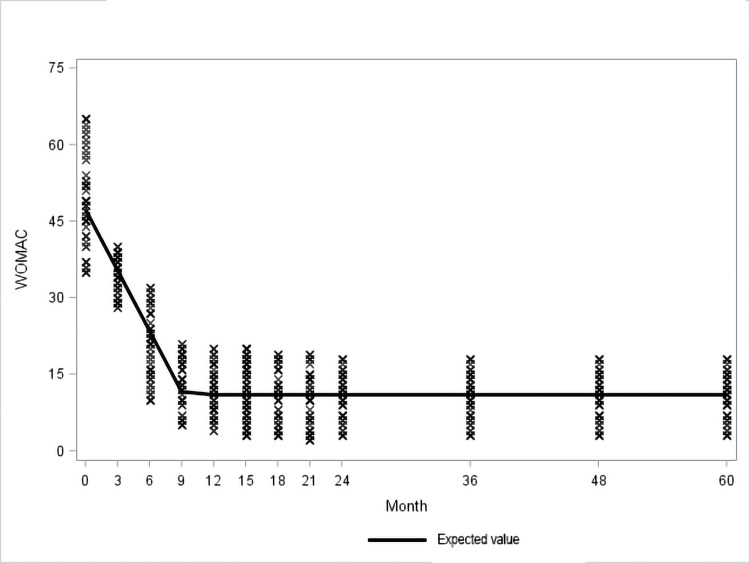
Observed and expected Western Ontario and McMaster Universities Arthritis Index (WOMAC) values

**Figure 4:**
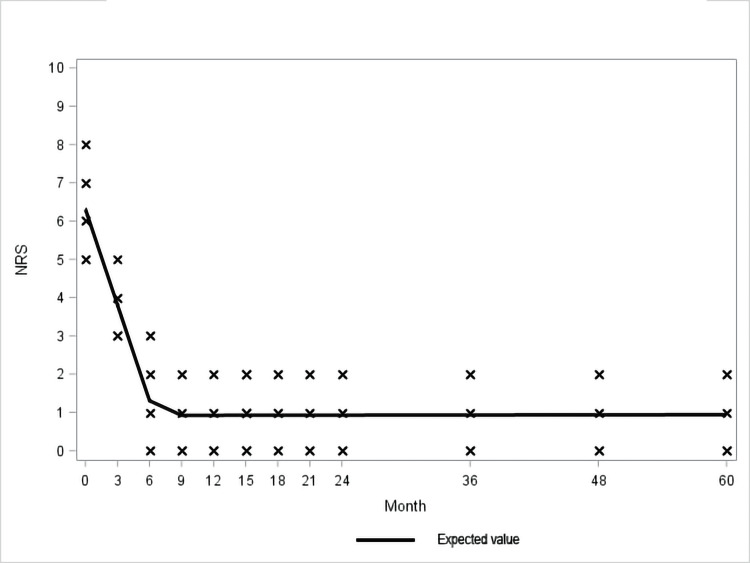
Observed and expected Numeric Rating Scale (NRS) values

The GEE linear regression model showed that the effect on knee pain and function of initial treatment supported by quarterly booster doses showed progressive benefit and appeared to reach its maximum potential between the sixth and ninth month when it stabilizes and remains virtually unchanged over time until the end of the study period (60 months) (Figure 3, 4; Table 3, 4).

**Table 3:** GEE linear regression model for the Western Ontario and McMaster Universities Arthritis Index (WOMAC) score GEE - Generalized Estimation Equation

Analysis of GEE parameter estimates						
QIC 786.9301						
QICu 784.0000						
Empirical standard error estimates						
Parameter	Estimate	Standard error	95% confidence limits		Z	Pr > Z
Intercept	47.2741	0.9623	45.3881	49.1601	49.13	< .0001>
Month	-3.9713	0.1501	-4.2655	-3.6770	-26.45	< .0001>
Month_9	-36.3520	1.0672	-38.4436	-34.2604	-34.06	< .0001>
Int_month_9	3.9716	0.1505	3.6767	4.2666	26.39	< .0001>

**Table 4:** GEE linear regression model for the Numeric Rating Scale (NRS) score GEE - Generalized Estimation Equation

Analysis of GEE parameter estimates						
QIC 786.2167						
QICu 784.0000						
Empirical standard error estimates						
Parameter	Estimate	Standard error	95% confidence limits		Z	Pr > Z
Intercept	6.3047	0.1183	6.0728	6.5366	53.29	< .0001>
Month	-0.8337	0.0361	-0.9045	-0.7630	-23.10	< .0001>
Month_6	-5.3853	0.1356	-5.6510	-5.1196	-39.72	< .0001>
Int_month_6	0.8340	0.0363	0.7628	0.9052	22.96	< .0001>

No major complications were observed; only five patients reported local pain in the injection site successfully treated with local ice application. None of them needed painkillers or non-steroidal anti-inflammatory drugs. No patients reported having any other specific knee treatments, including physiotherapy or the use of walking aids or braces. No pseudo-septic or septic arthritis was registered during the study period.

## Discussion

Intraarticular HA injection is a well-established treatment for symptomatic knee osteoarthritis. Hyalubrix® is a sterile nonpyrogenic solution of sodium hyaluronate (30 mg/2ml) with a molecular weight range of 1,500 to 3,200 kDa. It is produced by bacterial fermentation and has been used in the treatment of knee osteoarthritis with excellent results on pain reduction as well as functional improvement [16]. Moreover, ultrasound guidance increases accuracy and optimizes the efficacy of the intraarticular HA injection, and avoids collateral damage to nearby neurovascular structures. The accuracy under ultrasound guidance is 99% in comparison to the accuracy obtained by the landmark-guided technique, which is 79% [18].

To our knowledge, this is the first study evaluating the efficacy of the booster effect of a single injection of HA every three months (quarterly per year) with 60 months (five years) of treatment and follow-up in patients with low to moderate symptomatic knee osteoarthritis. The recorded variables, WOMAC score and NRS, significantly decrease, indicating an excellent and lasting response both in terms of pain and joint function.

The GEE linear regression model used in this study showed that, after the initial standard treatment with three-weekly injections, quarterly boosters allowed not only the achievement of the maximum effect in pain and joint function between the sixth and the ninth month of treatment but also this effect remained stable and unchanged until the completion of the five years of study. It is interesting to note that this trend is constant in all patients, regardless of the initial degree of osteoarthritis. Such significant long-term stable control of pain and improvement of function can be attributed to restoration of the viscoelasticity and maintenance of lubrication of the knee joint [19]. Also, it enhances chondrocytes' survival and the production of proteoglycans and hyaluronic acid. Additionally, it inhibits production and reduces levels of metalloproteinases, prostaglandins, interleukin-1β, nitric oxide, tumor necrosis factor-alpha, and increases levels of tissue inhibitor of metalloproteinase-1 [20]. HA also reduces lymphocyte motility and proliferation as well as inhibition of neutrophils' phagocytosis and degranulation [21]. The net result of the above-mentioned effects is the inhibition of inflammation and synovial hypertrophy. Also, HA acid reduces pain through a direct effect on nerves by modulating polymodal transient receptor potential vanilloid subtype 1 (TRPV1) channels, calcitonin gene-related peptide (CGRP), tyrosine receptor kinase A (TrkA) and acid-sensing ion channel 3 (ASIC3) [21-22] and this modulatory effects on joint afferents can be one of the mechanisms of the gap between HA residence time and duration of clinical efficacy. Additionally, it improves proprioception and isokinetic muscle force in patients with knee osteoarthritis [23].

The use of HA is associated with rare complications and long-term safety, as also confirmed by this study, which allows minimizing the use of NSAIDs and painkillers, burdened by major side effects (cardiovascular, gastrointestinal, kidneys, etc.), especially in elderly patients, and in those with comorbidities. Furthermore, previous studies had already demonstrated the non-superiority of single-dose treatments compared to the three-weekly standard protocol of HA viscosupplementation [9].

The choice to use this treatment protocol and to record its effects for such a long follow-up was based on the clinical observation and the benefits that the patients themselves reported, and the registers are one of the most appropriate tools for assessments of adequacy and effectiveness of an intervention. Although the registers, intended as a database of health services, indisputably present methodological biases, this is not the case. This study was inserted into a pre-existing ad-hoc registry dedicated to the treatment of painful musculoskeletal conditions (osteOArena), and the methodology and information collected are explicitly aimed at achieving the study aim, providing for actions (such as data collection and assessment scales) that go beyond the daily clinical practice, while not altering the substantial aspects (treatment). This data analysis has a clinical purpose, to evaluate the efficacy and safety of a treatment that had already entered clinical practice for years, even if based on evidence of efficacy that, until now, had to be considered inadequate.

Like all real-life registry-based studies, this study has limitations, mainly stemming from the lack of randomization, the absence of a control group or a placebo group, and the involvement of only a single center. Furthermore, the study did not include patients with K-L IV. The decision to exclude patients with grade IV KL was desired because the main purpose of this work was to evaluate the analgesic and functional effect of a booster dose of HA in the long term. The duration of the benefit of infiltrative treatments, of any kind, in severe gonarthrosis would plausibly not have been long, and this would have negatively distorted the results of the study. It would also have been very likely to see many dropouts due to more frequent complications, such as bone edema, subchondral fractures, osteonecrosis, or the need for prosthetic replacement.

The mean age of the patients may appear to be lower than expected but is probably justified by the inclusion of patients with symptomatic KL I / II and non-grade IV KOA. This finding could also, if not fully, be explained also by the origin of the patients. The patients included in this study refer to a specialized center of the first author dedicated for many years to the early diagnosis and treatment of musculoskeletal diseases, both degenerative and inflammatory. The mean BMI of these patients is also probably lower than expected, but they are generally patients with an active life and with an appropriate lifestyle and diet.

Being a study based on a single center, we cannot exclude that there is also a genetic or environmental influence on the population, although the clinical experiences of the authors, who come from different countries and continents, are similar when referred to the same conditions of this study.

The number of patients and the follow-up times are sufficient to establish results and significance, but a larger study population would increase the certainty of results.

## Conclusions

The quarterly intraarticular injection of HA for five years is a safe and effective treatment that alleviates pain and improves function in patients with mild to moderate knee osteoarthritis. This is the first evaluation of the use of long-term booster doses of HA, thus proposing it as a new therapeutic option in KOA. The reason that a quarterly injection of HA may achieve such a good and stable outcome for stage I~III OA knees might be that the effect span of a single HA injection may be less than, or no longer than three months, and the reason why we decided to do a quarterly injection is to "boost" the effect that otherwise would give the joint the potential time of exposure to wear and tear. The results observed, therefore, seem to support this hypothesis. Further randomized controlled trials with a larger number of patients or multicentric are needed to confirm these findings. 
